# A Synbiotic for the Oral–Nasal Gateway: The Mechanistic Rationale, Pathogen-Targeting Profile, and Implications for Gut and Pulmonary Health

**DOI:** 10.3390/biomedicines14102268

**Published:** 2026-10-07

**Authors:** John N. Peldyak, Paul Reynolds, César Alas Pineda, Gustavo Ferrer, Mark L. Cannon

**Affiliations:** 1 Independent Researcher, Mount Pleasant, MI 48858, USA; 2 Department of Cell Biology and Physiology, Brigham Young University, Provo, UT 84602, USA; 3 Department of Research and Development, Moxie Health Group, Hallandale Beach, FL 33008, USA; calas@moxie-health.com; 4 Pulmonary and Critical Care Medicine, Aventura Hospital and Medical Center, Aventura, FL 33180, USA; gferrer@pulmonary-institute.com; 5 Northwestern University Feinberg School of Medicine, Chicago, IL 60611, USA; 6 Ann & Robert H. Lurie Children’s Hospital of Chicago, Chicago, IL 60611, USA

**Keywords:** oral microbiome, nasal microbiome, oral–nasal gateway, synbiotic, *Streptococcus salivarius* M18, xylitol, oral–gut axis, gut–lung axis, oral–systemic health, probiotics

## Abstract

The oral cavity and the nasal passage form a single, continuously mixed ecological gateway rather than two independent niches: saliva is swallowed roughly once every one to two minutes while awake, and nasal mucus is transported posteriorly into the nasopharynx on a cycle of roughly ten to twenty minutes, so oral and nasal microbial communities are in near-constant fluid exchange with each other and with the gut. This narrative review examines the mechanistic rationale for a proposed formulation containing *Streptococcus salivarius* M18, *Lactobacillus plantarum* HEAL9, *Lacticaseibacillus paracasei* 8700:2, and *Bifidobacterium longum* subsp. *infantis*, with 2 g/day xylitol and vitamins C and D3. We assess component-level evidence for bacteriocin-mediated antagonism, immune modulation, colonization resistance, and airway-surface effects and relate these findings to oral–gut translocation, lung microbiome seeding, and gut–lung immune signaling. The strongest clinical support concerns caries-related and upper respiratory outcomes for individual components or strain pairs. Gut effects depend on strain identity, age, diet, and substrate availability; pulmonary benefits remain indirect hypotheses. No trial has tested the complete formulation, and evidence from topical nasal xylitol cannot establish nasal efficacy after oral administration. Cardiovascular findings involving circulating xylitol and experimental platelet activation warrant dose-specific safety evaluation; their relevance to 2 g/day remains uncertain. Trials should establish strain identity, compatibility, viability, and safety, then pair clinical endpoints with longitudinal oral, nasal, and intestinal sampling. The proposed formulation offers a testable approach to the oral–nasal–gut–pulmonary axis, but its efficacy and safety require product-specific confirmation.

## 1. Introduction

The mouth is the shared entrance to the digestive tract and the upper airway. Organisms, dietary substrates, and host secretions are all present at this interface before they reach the gut, the lungs, or the circulation [1]. This connectivity is not occasional: as detailed in Section 3.1, the oral and nasal compartments exchange fluid and microorganisms on a timescale of minutes, through swallowing and nasal mucociliary clearance, and are therefore best modeled as a single, dynamically mixed ecosystem rather than as two adjacent but separate niches. A supplement designed for this interface should act across multiple compartments accordingly. The formulation described in this review pairs four evidence-supported bacterial strains with xylitol and two immune-active vitamins to address cariogenic, periodontal, respiratory, and enteric targets simultaneously and extends the analysis to the gut and pulmonary consequences of gateway ecology (Section 9).

*Streptococcus salivarius* M18 exerts bacteriocin-mediated antagonism against cariogenic and periodontal organisms, as well as against *Streptococcus pyogenes* [2,3]. *Lactobacillus plantarum* HEAL9 and *Lacticaseibacillus paracasei* 8700:2 are an immune-modulating pair that has been studied for reducing common cold burden [4,5]. *Bifidobacterium longum* subsp. *infantis* is a specialist infant-gut colonizer that supports colonization resistance and early immune imprinting [6,7]. Xylitol at 2 g interferes with mutans streptococci and, as an airway osmolyte, reduces bacterial adhesion in the nasal passages [8,9]. Vitamin C and vitamin D3 support mucosal and innate immunity, with vitamin D3 inducing the antimicrobial peptide cathelicidin [10,11].

The practical endpoint is not sterilization but restoration of microbial homeostasis across the oral–nasal–gut–pulmonary continuum. By reducing the oral inflammatory reservoir, limiting the flow of pathobionts toward the airway and gut, and supporting mucosal colonization resistance, the formulation may also influence upstream pathways relevant to respiratory and gastrointestinal health and, more speculatively, to cardiometabolic outcomes. These are potential oral–systemic benefits and require product-specific confirmation; they are not treatment claims for systemic disease. Figure 5 (Section 8) shows how each component maps to a compartment and to a health outcome.

Interest in the mouth as a determinant of systemic health has grown with improved sampling and sequencing methods. The oral microbiome is now described as a set of spatially organized communities whose composition differs by site and shifts in characteristic ways in caries and periodontitis [12,13]. Experimental work has also identified routes by which periodontal inflammation can affect distant tissues, including dissemination of oral bacteria and their products, systemic cytokine exposure, and inflammatory reprogramming of bone-marrow progenitors [14]. These findings have moved oral–systemic research from association toward testable mechanisms, although intervention trials with systemic endpoints remain few.

A similar change has occurred in respiratory biology. Researchers now study the anterior nares, nasopharynx, and oropharynx as connected microbial habitats that influence susceptibility to respiratory infection [15]. In the lower airway, enrichment with oral taxa such as *Prevotella* and *Veillonella* has been associated with a T helper 17 cell inflammatory profile in asymptomatic adults [16], and exchange between oral and lung communities is now considered relevant to pneumonia, chronic obstructive pulmonary disease (COPD), and other lung disorders [17]. These observations support examining the oral and nasal compartments together rather than separately.

Microbiome-based therapeutics have matured over the same period. Consensus definitions now distinguish probiotics, prebiotics, and synbiotics [18], and the clinical literature increasingly treats benefit as strain-, dose-, and indication-specific rather than as a property of probiotics as a class [19]. A trial of a purified bacterial consortium for recurrent *Clostridioides difficile* infection, followed by regulatory approval, showed that defined microbial products can be developed and tested to pharmaceutical standards [20]. Under the current consensus, a synbiotic may be complementary, with each component acting independently, or synergistic, with a substrate selected for use by the co-administered organisms [21]. The formulation discussed here is complementary: xylitol and the vitamins are not intended as selective substrates for the included strains, and each ingredient carries its own rationale.

This review therefore has a narrower objective than a general survey of oral–systemic health. It asks whether the published evidence supports the rationale for one oral–nasal synbiotic, what that evidence shows for each component, and where the gaps lie between component-level findings and claims for the finished product. We include gut, pulmonary, and systemic pathways only as they bear on this question, and we grade each proposed benefit by level of evidence (Table 5 and Figure 6).

## 2. Materials and Methods

### 2.1. Search Strategy

This structured narrative review used PubMed/MEDLINE and Google Scholar from database inception through 1 September 2026. We searched reference lists of eligible articles and relevant reviews and used PubMed and publisher records to verify bibliographic details and digital object identifiers. We updated the search to add recent work on oral and respiratory microbiome biology, microbiome-based therapeutics, and probiotic trial methodology.

Search terms were combined in groups covering: (i) the oral, nasal, nasopharyngeal, oropharyngeal, gut, and lung microbiomes; (ii) *Streptococcus salivarius* M18, *Lactobacillus plantarum* HEAL9, *Lacticaseibacillus paracasei* 8700:2, *Bifidobacterium longum* subsp. *infantis*, xylitol, vitamin C, and vitamin D3; (iii) caries, periodontitis, otitis media, rhinosinusitis, respiratory infection, microbial translocation, microaspiration, gut-lung signaling, cardiovascular disease, diabetes, platelet activation, thrombosis; and (iv) probiotic, prebiotic, synbiotic, bacteriocin, colonization resistance, immune modulation, and mucosal barrier. Searches used former bacterial nomenclature, Boolean operators, and citation chaining.

### 2.2. Eligibility and Evidence Selection

Eligible sources included peer-reviewed human trials, observational studies, systematic reviews and meta-analyses, consensus reports, and mechanistic studies in animals, tissues, cells, or microbial systems when they directly informed a formulation component or an oral–nasal–gut–pulmonary pathway. English-language full texts were prioritized. Reports were excluded when they were duplicative, lacked sufficient methodological detail, did not identify the probiotic strain for a strain-specific claim, or did not address a prespecified organism, ingredient, compartment, mechanism, safety outcome, or clinical endpoint. Conference abstracts and non-peer-reviewed reports were not used to support efficacy claims. Commentaries were retained only when they directly addressed interpretation of the published xylitol cardiovascular safety signal.

### 2.3. Evidence Classification and Synthesis

Titles and abstracts were screened for relevance, followed by full-text review when strain identity, dose, route, comparator, mechanism, or outcome required confirmation. Findings were synthesized narratively because the interventions and endpoints were too heterogeneous for pooled analysis. Evidence was classified as direct finished-product evidence, component- or strain-specific clinical evidence, mechanistic or translational evidence, or no direct evidence. Statements were limited to the level supported by the underlying design; evidence from one strain, dose, route, age group, or anatomical compartment was not treated as proof for another. Table 4 defines the evidence tiers, and Table 5 and Figure 6 apply them to each proposed benefit.

## 3. The Oral–Nasal Gateway: A Continuous Ecosystem

The oral cavity contains one of the body’s most diverse microbial ecosystems, distributed across the tooth, gingival, tongue, palatal, tonsillar, and salivary niches. Because the mouth is both the start of the digestive tract and a functional part of the upper airway, it is best understood as a gateway ecosystem rather than an isolated dental compartment [1]. The nasal microbiome is a second airway gateway. In health, it is dominated by *Corynebacterium*, *Dolosigranulum*, *Cutibacterium*, and *Staphylococcus epidermidis*; in disease, it shifts toward *Staphylococcus aureus*, *Haemophilus influenzae*, *Moraxella catarrhalis*, and *Streptococcus pneumoniae* [1,22].

These compartments are continuous. The nasopharynx connects the nasal and oral communities; saliva is swallowed continuously, and a dysbiotic nasal passage can seed pathobionts into the oropharynx and, through swallowing, into the gut [1,22]. A single-target intervention is therefore poorly matched to the biology. A product that combines direct bacterial antagonism, immune modulation, colonization resistance, and airway-active chemistry is better aligned with the gateway’s connected structure. The infant and maternal literature reinforces this logic: nasopharyngeal ecology in early life predicts the severity of lower respiratory infection and the risk of asthma [22].

### 3.1. Temporal and Fluid-Dynamic Basis of Oral–Nasal Overlap

The continuity described above is not a static anatomical fact; it is renewed by fluid movement on a time scale of minutes. Two independent physiological clocks keep the oral and nasal compartments in near-constant contact. The first is swallowing. Healthy adults swallow saliva about once every one to two minutes while awake, a rate first quantified systematically by Lear and colleagues, with swallowing frequency falling by roughly an order of magnitude during sleep [23]. Each swallow moves a mixed bolus of salivary and posterior nasal secretions across the oropharynx and into the esophagus, so the mouth is never a closed system for more than a few minutes at a time while a person is awake.

Nasal mucociliary clearance continuously transports mucus toward the nasopharynx, where it is swallowed with saliva. Classical clearance studies report transit times of approximately 10–20 min in healthy adults [24]. Transit time describes the movement of mucus across the nasal surface, rather than a discrete cycle of microbial exchange.

**Figure 1:**
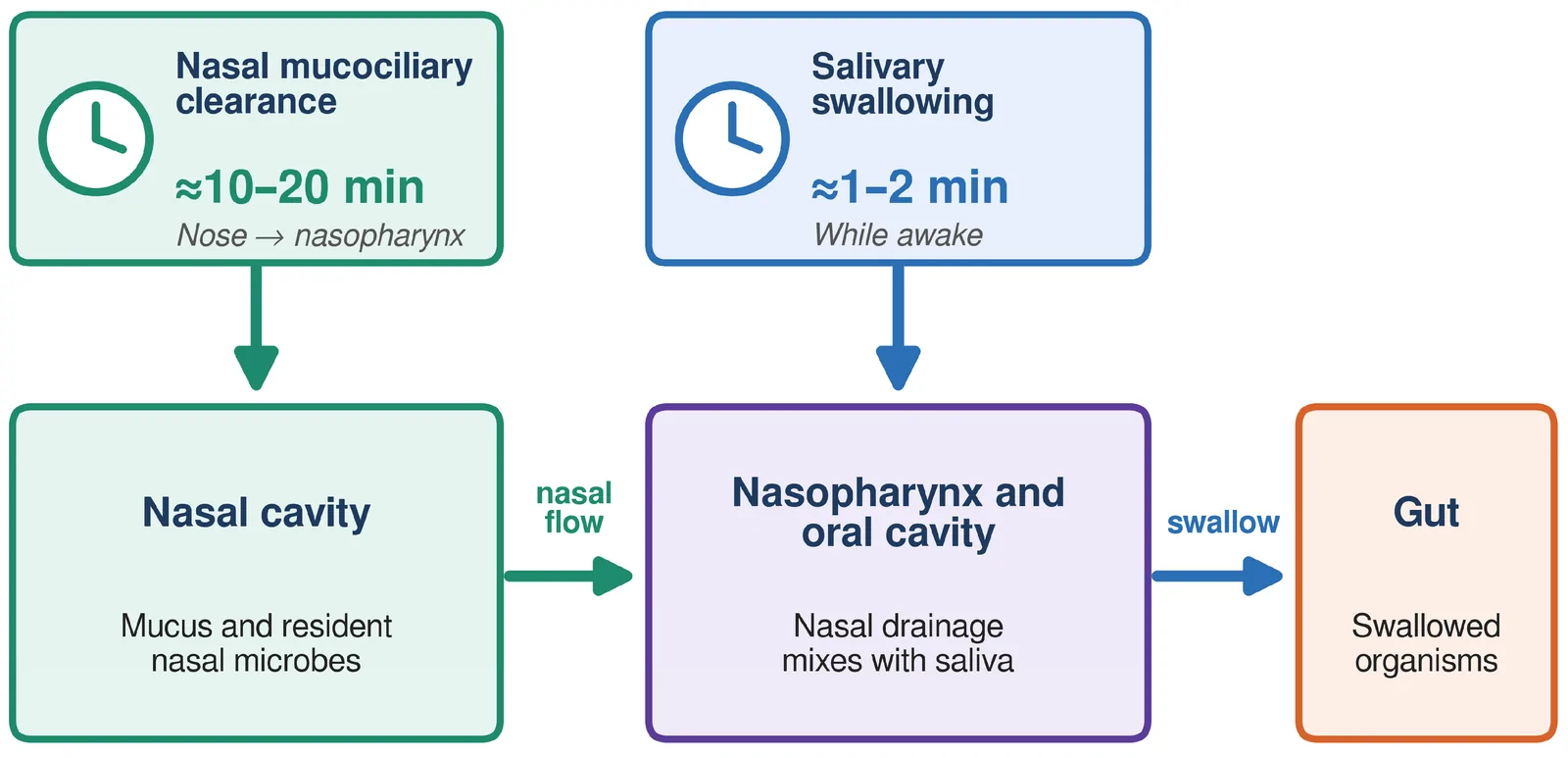
Mucociliary clearance and swallowing link the oral, nasal, and gut compartments. Nasal mucociliary clearance delivers nasal secretions to the nasopharynx, where they are swallowed with saliva, with a transit time of approximately 10–20 min [24]; independently, saliva itself is swallowed roughly once every one to two minutes while awake [23]. Together, these two clocks keep the oral, nasal, and gut compartments in near-continuous fluid and microbial exchange.

This is not merely an anatomical argument; it is reflected in microbial community structure. In a culture-independent survey of the healthy adult nasal cavity, nasal bacterial communities resembled oral communities more closely than they resembled skin communities from the same individuals, a pattern the authors attributed to posterior nasal drainage and its continuous mixing with the oropharynx [25]. More broadly, the respiratory tract has been framed as a single, anatomically continuous ecosystem, in which the nasopharynx functions as the central transitional niche linking the nasal cavity, the oral cavity and oropharynx, and the lower airway, rather than as a boundary between separate microbial territories [26]. Figure 1 summarizes the two clocks and the resulting continuous exchange among the nasal, oral, and gut compartments.

These physiological connections support testing whether an oral intervention changes the oropharyngeal reservoir and downstream gut exposure. They do not establish that an orally delivered formulation reaches the nasal mucosa or modifies nasal ecology; those effects require route-specific testing.

## 4. Formulation Overview

Table 1 summarizes each component, a representative daily amount, the primary compartment addressed, and the principal documented role. Doses shown are typical of those reported in the published literature and in marketed formulations; the proposed finished-product specifications govern label claims.

## 5. Component Monographs

### 5.1. Streptococcus salivarius M18

*S. salivarius* is an early and abundant colonizer of the healthy oral cavity and oropharynx. Strain M18 produces bacteriocin-like inhibitory substances, including salivaricin A2 and salivaricin 9, together with dextranase and urease activities that counter plaque accumulation and acidification [3]. In a randomized, double-blind, placebo-controlled trial in children, M18 improved plaque-related indices, with the greatest benefit in those with the highest baseline plaque and cariogenic burden [2]. Cariogram-based evaluation reported improved caries-risk profiles after 90 days of administration [27]. A later randomized clinical trial also reported reduced salivary *S. mutans* among preschool children receiving M18 [28]. M18 is therefore more than a generic oral probiotic: its proposed value rests on strain-specific bacteriocin production, interference with extracellular polysaccharide structure, and support of a less acidogenic plaque environment.

Because *S. salivarius* colonizes the oropharynx, its bacteriocin activity is relevant to the shared nasal–oral airway. Lactobacilli and salivarius-type organisms have demonstrated colonization-resistance activity against respiratory pathogens, and delivery routes that reach the oropharynx plausibly contribute to airway defense [29].

### 5.2. Lactobacillus plantarum HEAL9

HEAL9 is a well-characterized immune-modulating strain. In a randomized, double-blind, placebo-controlled trial, the combination of *L. plantarum* HEAL9 and *L. paracasei* 8700:2 reduced the incidence and duration of common cold episodes and altered circulating immune cell responses [4]. A subsequent controlled study reported reduced frequency and severity of cold symptoms with the same lactobacilli [5]. A 2022 Cochrane review of probiotics for acute upper respiratory tract infections included this strain pair among the commonly studied preparations and found that probiotics as a class may reduce the number of people experiencing infection and may shorten episode duration, although certainty varied by outcome and the results cannot be transferred automatically from one strain to another [30]. The HEAL9/8700:2 evidence is best interpreted as host-mediated support of mucosal defense rather than direct killing of respiratory viruses.

### 5.3. Lacticaseibacillus paracasei 8700:2

8700:2 is the companion strain to HEAL9 in the immune-support literature and is reported under the reclassified genus *Lacticaseibacillus*. Researchers have studied the pair together in upper respiratory outcomes, where their combination, rather than either strain alone, shows the reported benefit [4,5]. Both strains contribute to gut-level colonization resistance and to the gut–airway immune axis that links intestinal probiotic exposure to respiratory outcomes [31].

### 5.4. Bifidobacterium longum *subsp.* infantis

*B. infantis* is the archetypal specialist colonizer of the breastfed infant gut, equipped to import and metabolize a broad range of human milk oligosaccharides (HMOs) [6]. Supplemented *B. infantis* EVC001 can persist in breastfed infants when the appropriate milk substrate is available, shifting the community toward bifidobacterial dominance and away from organisms that preferentially consume simpler carbohydrates or host-derived substrates [32]. This ecological change is accompanied by lower fecal pH, altered metabolite production, and reduced markers of intestinal inflammation [7,32]. Bifidobacteria-mediated signals also participate in early immune imprinting [7]. These findings support barrier integrity and colonization resistance, but they are strain-, age-, diet-, and substrate-dependent. Evidence from EVC001 or other *B. longum* strains should not be presented as direct proof for an unspecified *B. infantis* ingredient unless the formulation identifies and validates its strain.

### 5.5. Clinical Evidence from Oral-Probiotic Studies

Probiotic publications provide a clinical bridge between the general probiotic literature and the oral-gateway formulation developed in this review. The 2011 review proposed selecting and evaluating oral probiotics as ecological interventions, using microbial testing to identify the dominant risk pattern and track the response. The purpose was not indiscriminate supplementation, but the deliberate use of beneficial organisms to modify a dysbiotic oral biofilm while preserving commensal function [33]. That framework remains directly relevant to a multi-strain oral–nasal synbiotic.

A pediatric study evaluated caries-prone children receiving oral probiotic therapy and followed salivary mutans streptococci and lactobacilli with the Caries Risk Test (CRT). The study demonstrated that salivary microbial monitoring could detect changes during probiotic use and could be incorporated into caries-risk management [34]. The later retrospective clinical review examined 60 patients enrolled in an oral probiotic program and reported a statistically significant favorable effect on caries experience [35]. These studies support continued clinical investigation of oral probiotics, but they do not establish that every commercial strain, dose, or combination produces the same outcome.

Together, probiotic studies support three practical principles. First, probiotic effects are strain-specific and can be site-specific. Second, sustained exposure is more biologically credible than a brief course because oral colonization and community restructuring take time. Third, salivary testing should accompany clinical indices so that reductions in caries or gingival risk can be linked to measurable ecological changes [33,34,35].

### 5.6. Xylitol (2 g)

Xylitol is a five-carbon polyol that mutans streptococci take up but cannot ferment to acid, producing a futile intracellular cycle that suppresses growth and reduces adhesion and transmissibility [8]. A dose–response study established that repeated small daily amounts reduce salivary mutans streptococci [36]. Maternal xylitol use reduces mother-to-child transmission of mutans streptococci, a finding confirmed by meta-analysis [37,38]. In an optical-density study, xylitol produced concentration-dependent inhibition of *S. mutans*, *S. anginosus*, and *F. nucleatum*, while *C. albicans* was comparatively resistant under the tested conditions [39]. The findings demonstrate taxon-specific activity in planktonic screening and support follow-up in polymicrobial biofilms; they should not be read as proof of clinical success. Xylitol also acts against *P. gingivalis*: it inhibited inflammatory cytokine expression induced by *P. gingivalis* lipopolysaccharide [40] and inhibited the organism and the inflammatory response of human macrophages infected with it [41]. Beyond the mouth, xylitol lowers the salt concentration of airway surface liquid, supports endogenous antimicrobial activity, and reduces growth or adhesion of selected nasopharyngeal pathogens [9,42,43,44,45].

### 5.7. Vitamin C

Vitamin C supports phagocyte function and epithelial barriers and is required for collagen synthesis, which underlies gingival integrity. Meta-analysis shows that regular supplementation modestly shortens the duration of common cold episodes, with larger effects under physiological stress [10]. Micronutrient status, including vitamin C, is linked to periodontal health and to the resolution of gingival inflammation [46]. In this formulation, vitamin C acts as a host-support ingredient rather than a direct antimicrobial and is represented accordingly in Figure 3.

### 5.8. Vitamin D3

Vitamin D3 modulates both innate and adaptive immunity. The vitamin D receptor directly upregulates the human cathelicidin antimicrobial peptide gene, linking vitamin D status to endogenous antimicrobial capacity at mucosal surfaces [11]. A large individual-participant data meta-analysis found that vitamin D supplementation reduces the risk of acute respiratory tract infection, with the greatest benefit in those most deficient [47]. Vitamin D status is also associated with periodontal outcomes [48]. Together with vitamin C, vitamin D3 supplies the host-defense arm that complements the direct and ecological actions of the probiotic strains and xylitol.

## 6. Pathogen-Targeting Profile: An Integrated View

**Figure 2:**
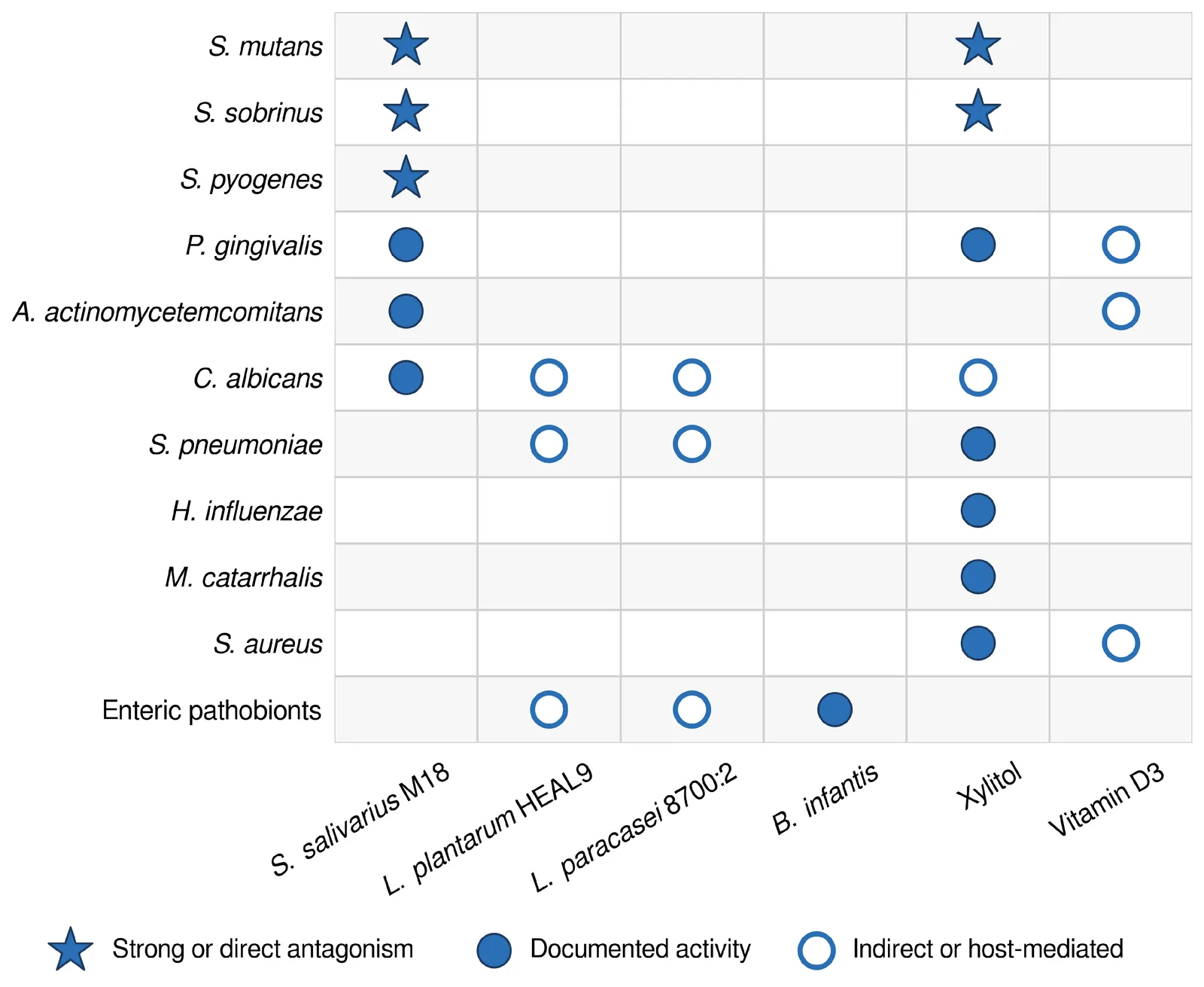
Documented component–pathogen activity matrix. Markers reflect the direction and directness of published in vitro, ex vivo, or clinical evidence for each pairing. Open circles denote benefit through host immune or ecological mechanisms rather than direct effect. Vitamin C is not shown because no organism-specific activity was identified; its contribution is host-mediated (Figure 3).

The rationale for combining the components is clearest when they are considered together against the pathogens that matter across the oral and nasal gateway interface. Figure 2 maps each component to eleven clinically relevant organism groups, spanning cariogenic, periodontal, nasal/respiratory, and enteric categories. The categories are ordinal and mechanism-grounded: a star denotes strong or direct antagonism, a filled circle denotes documented activity, and an open circle denotes an indirect or host-mediated benefit. The categories describe the direction and directness of published evidence; they are not pooled effect sizes or head-to-head potencies.

Two patterns stand out. First, direct antagonism is concentrated in *S. salivarius* M18 (cariogenic, pyogenic and periodontal pathogens) and xylitol (mutans streptococci, *P. gingivalis*, and the nasal respiratory quartet). Second, the lactobacilli, *B. infantis*, and vitamin D3 provide breadth through host-mediated and ecological mechanisms, extending coverage to organisms that no single component kills outright. Table 2 restates these relationships, pathogen by pathogen.

**Figure 3:**
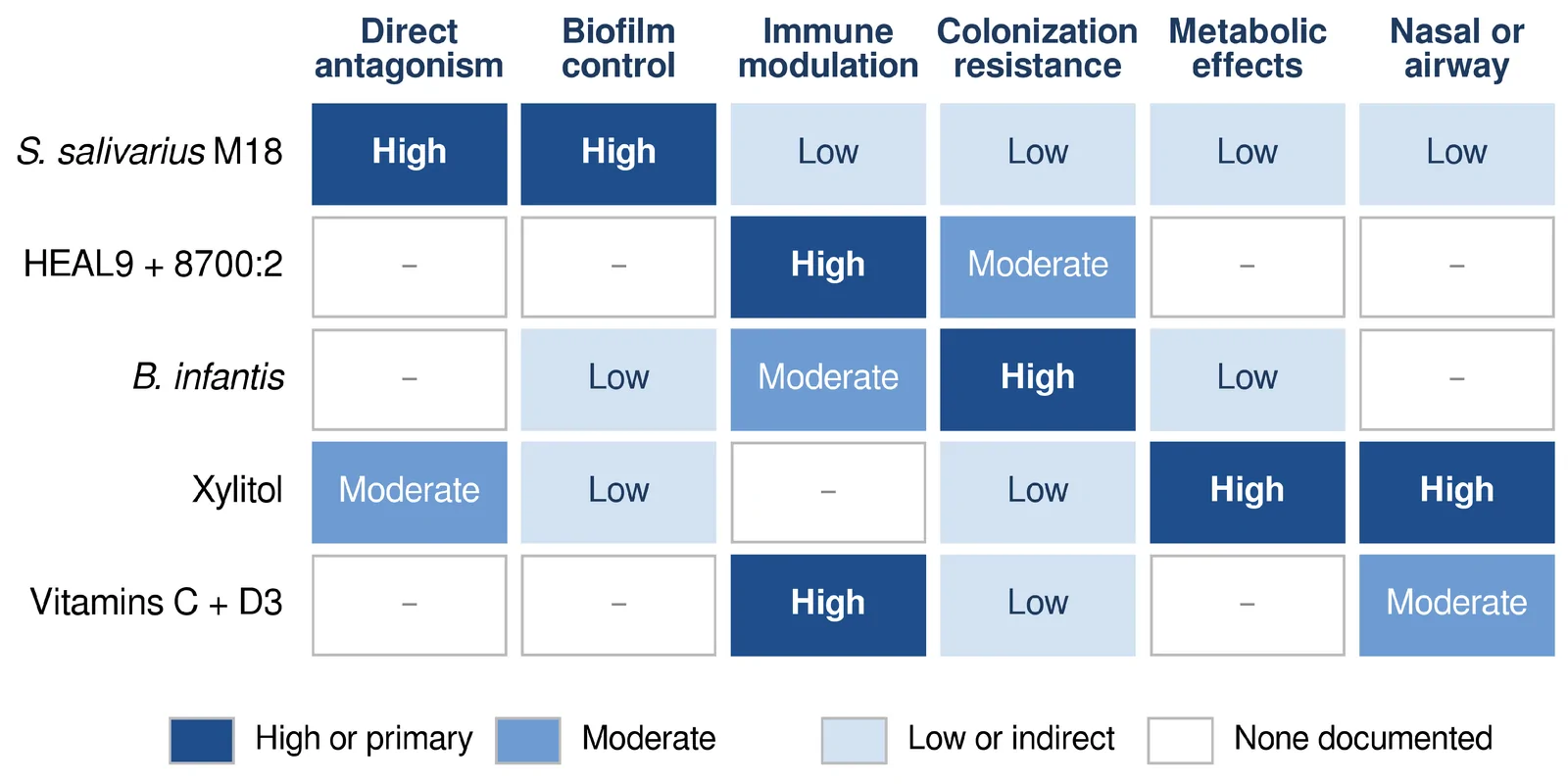
Mechanistic contribution profiles by component class. Cell shading indicates a qualitative high, moderate, or low emphasis across six modes of action; a dash indicates no documented contribution. Ratings are not pooled effect-size estimates.

Figure 3 recasts the same evidence as mechanistic contribution profiles. *S. salivarius* M18 leads in direct antagonism and biofilm interference; the lactobacilli and *B. infantis* lead in immune modulation and colonization resistance; xylitol leads in metabolic reprogramming and nasal activity. These complementary profiles support combining these components rather than using any one alone.

## 7. The Nasal Gateway and the Shared Upper-Airway Reservoir

Conventional care has underserved the oral/nasal gateway, favoring pharmaceutical and surgical management of rhinosinusitis and allergic airway disease over microbiome restoration [49]. Principles developed for oral probiotic therapy provide a rationale for investigating nasal ecological interventions [49]. Lactobacilli occupy a niche in the human nose and can antagonize selected respiratory pathogens [29], but effects must be established for the specific strain and delivery route.

Continuous mucociliary drainage and swallowing link nasal and oropharyngeal communities [25,26]. This connectivity does not eliminate site-specific differences in community composition or colonization resistance.

Xylitol adds a distinct nasal mechanism. As an osmolyte, it lowers the salt concentration of the airway surface liquid, thereby enhancing the activity of endogenous antimicrobial peptides and reducing bacterial adhesion to the mucosa [9]. In vitro, xylitol inhibits the growth of nasopharyngeal bacteria, including *S. pneumoniae*, in the presence of common dietary sugars [44,45]. These are the same organisms that dominate nasal dysbiosis and that feed forward into oral and gastrointestinal colonization, which is why a nasal benefit is not confined to the nose [1].

**Figure 4:**
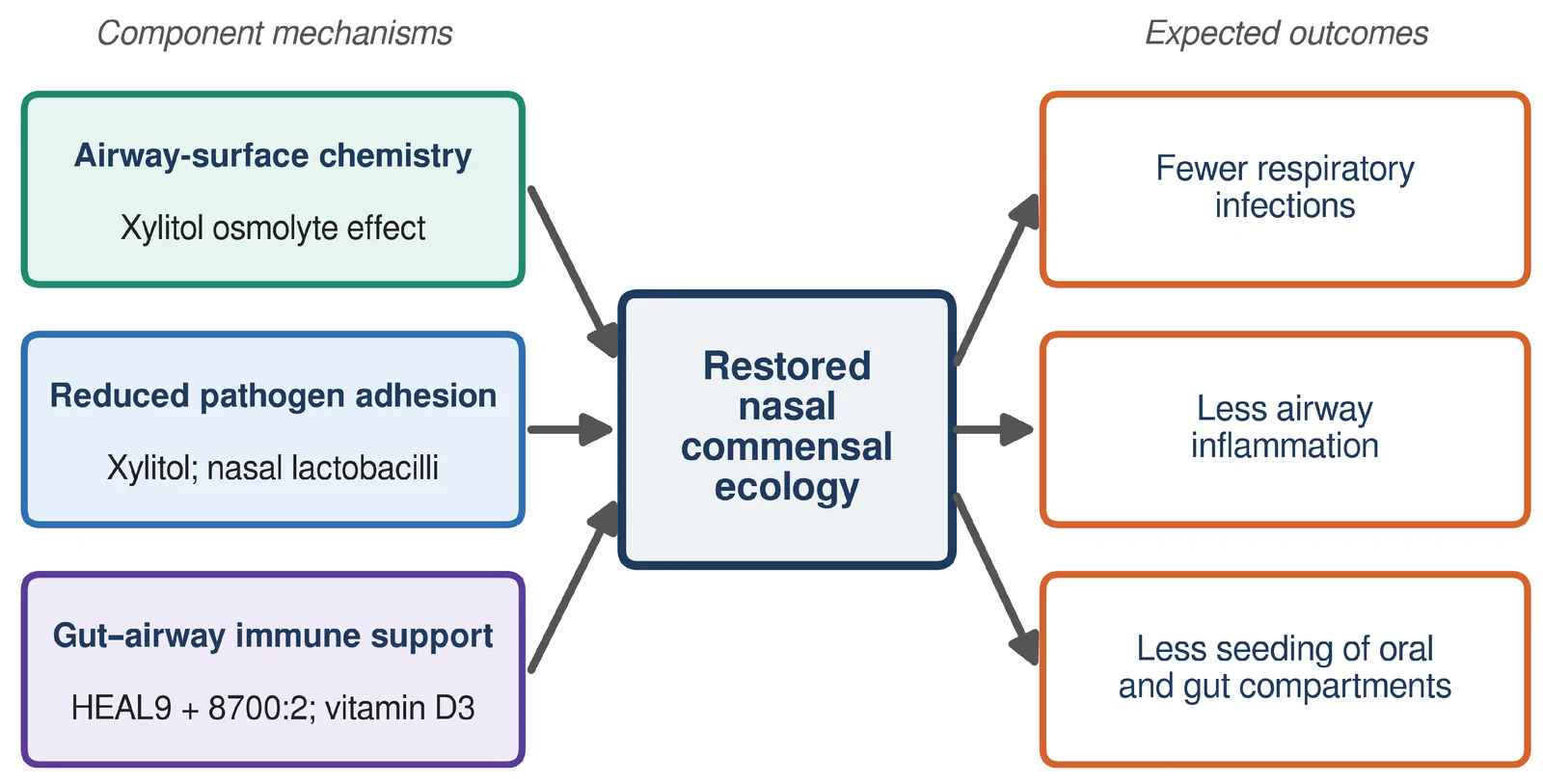
Proposed nasal gateway pathways. Airway-surface effects of topically applied xylitol and probiotic-associated mucosal immune mechanisms provide a rationale for testing changes in nasal ecology and respiratory outcomes. These effects have not been demonstrated for the complete oral formulation.

A third, indirect pathway operates through the gut–airway immune axis. Probiotic exposure in the gut is associated with reduced upper respiratory infection and lower allergic sensitization, outcomes consistent with improved systemic mucosal immune programming rather than direct nasal colonization [31]. Figure 4 shows how these three pathways, airway-surface chemistry, reduced pathogen adhesion, and gut–airway immunity, could influence nasal ecology, respiratory infection burden, airway inflammation, and downstream seeding of the oral and gut compartments.

Practical note on delivery: An oral synbiotic could influence nasal outcomes principally through the oropharyngeal reservoir and the gut–airway axis. Direct nasal application of xylitol, by isotonic spray or irrigation, is a complementary route that reaches the mucosa more directly and is supported by the airway-surface literature [9]. Their combined effects and tolerability require direct evaluation.

## 8. Complementary Mechanisms and the Multi-Compartment Model

**Figure 5:**
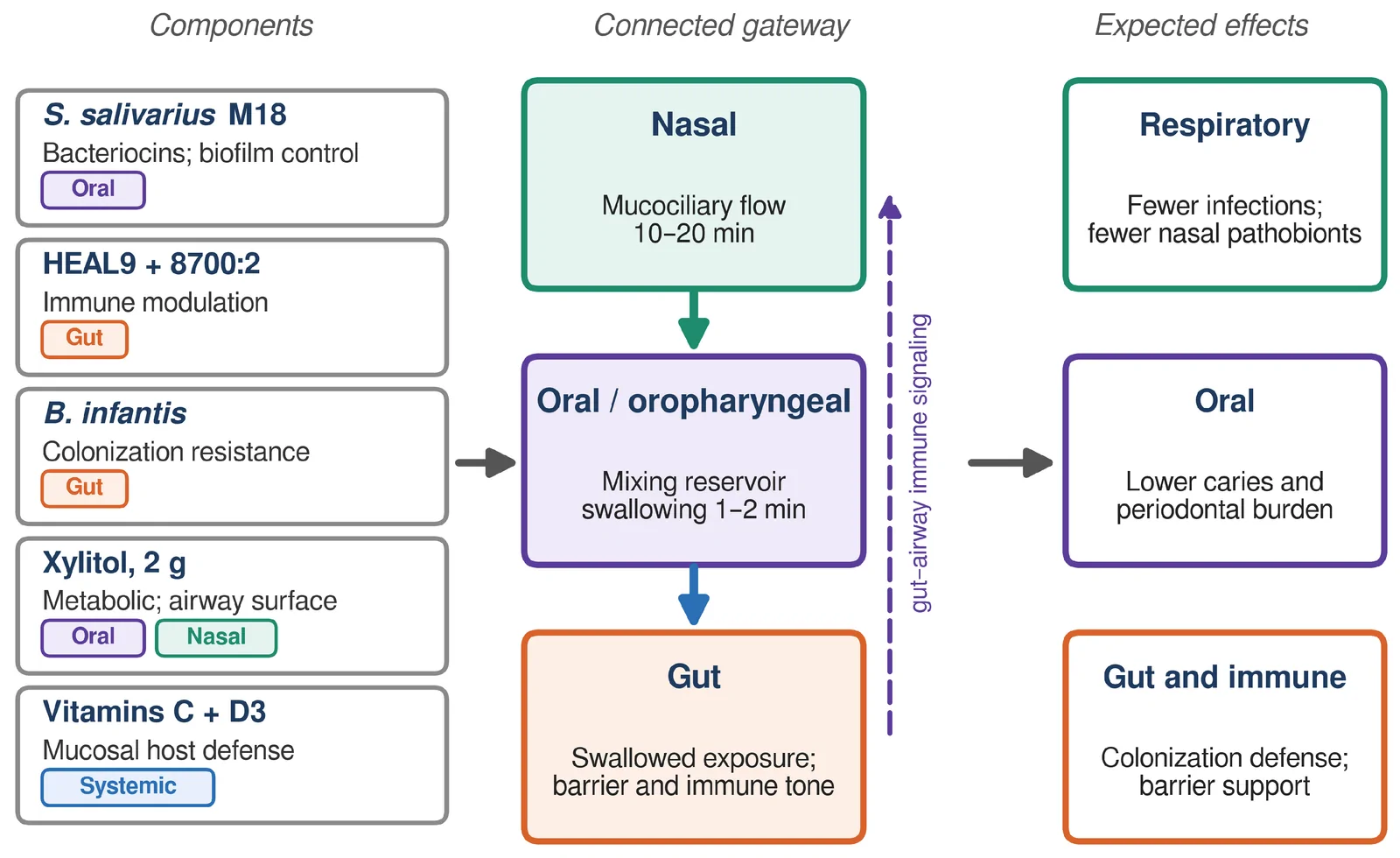
Multi-compartment gateway model. Components act through complementary mechanisms across connected nasal, oral/oropharyngeal, and gut niches; aggregate effects require testing in trials of the finished formulation.

Figure 5 integrates the formulation into a single model. On the left, each component is linked to the compartment or compartments it acts on. In the center, the nasal, oral, and gut compartments exchange organisms through the nasopharynx and through continuous swallowing, on the minute-scale cycles described in Section 3.1. On the right, the hypothesized outcomes are fewer respiratory infections and reduced nasal pathobionts, a lower cariogenic and periodontal burden, and stronger colonization resistance with early immune imprinting.

The proposed complementarity follows the routes connecting these compartments: oral antagonism may reduce organisms entering the gut or lower airway, while gut-mediated immune effects may influence respiratory defenses. Whether the combination improves outcomes beyond its individual components remains unknown. Interactions among strains may be additive, neutral, or antagonistic (Section 12.2).

## 9. Potential Benefits Across the Oral–Nasal–Gut–Pulmonary Axis

The rationale begins with the oral reservoir. Periodontitis can increase exposure to oral organisms and inflammatory products through swallowing, transient bacteremia, and systemic signaling [50]. Reducing this burden while supporting intestinal colonization resistance could influence downstream gut and airway exposure, but this remains a hypothesis for the proposed formulation.

### 9.1. Periodontal Inflammation as the Modifiable Upstream Target

The most immediate systemic opportunity is reducing the inflamed oral reservoir. *P. gingivalis*, *F. nucleatum*, and *A. actinomycetemcomitans* can stimulate gingival fibroblasts and epithelial cells to release interleukin (IL)-6, IL-8, and other mediators that recruit and activate inflammatory cells. In an experimental gingival fibroblast model, *S. salivarius* suppressed cytokine activation induced by these periodontal pathogens and altered the salivary community without acting as a broad-spectrum antiseptic [51]. This is a useful distinction. A commensal probiotic is intended to reshape signaling and ecological competition while preserving the resident community, rather than sterilizing the mouth.

Clinical meta-analyses of oral probiotics used with nonsurgical periodontal therapy report modest improvements in probing depth, clinical attachment, bleeding, or selected microbial outcomes in some populations, but heterogeneity remains substantial [52,53,54]. These findings suggest that selected probiotic preparations may improve local periodontal control when used with professional debridement and daily plaque control. It does not replace diagnosis, scaling and root planing, treatment of active infection, or maintenance care. If the formulation lowers bleeding and the abundance or inflammatory activity of periodontal pathobionts, the frequency and magnitude of microbial and inflammatory spillover should also fall. This downstream effect is plausible and measurable, but formulation-specific trials should confirm it using periodontal indices, high-sensitivity C-reactive protein, IL-6, endotoxin-related markers, and salivary or plasma microbial DNA.

### 9.2. Systemic Context and Xylitol Safety

Periodontitis is associated with atherosclerotic cardiovascular disease through overlapping inflammatory risk, bacteremia, endothelial activation, altered lipid handling, and direct detection of oral organisms or their products in vascular tissues. A joint periodontal-cardiology consensus concluded that the epidemiologic association is consistent and biologically plausible, while also emphasizing that periodontal treatment has not been proven to prevent myocardial infarction or stroke [55]. The formulation’s potential benefit is therefore upstream: improved gingival health may reduce inflammatory exposure and oral pathogen burden, while xylitol replaces fermentable sugar and the probiotic components support a less dysbiotic oral and intestinal ecology.

Separate from oral-inflammatory pathways, a cardiovascular safety concern for xylitol requires cautionary consideration. Witkowski et al. reported that fasting plasma xylitol in a cardiovascular referral population was associated with incident three-year major adverse cardiovascular events in an independent validation cohort; the adjusted hazard ratio for the highest versus lowest tertile was 1.57 (95% confidence interval, 1.12-2.21) [56]. Complementary studies using human platelets, platelet-rich plasma, whole blood, and mouse thrombosis models showed increased platelet responsiveness or thrombus formation. In a small acute intervention, ten healthy volunteers also showed increased platelet-function measures after drinking 30 g of xylitol within a 2-min period, a single dose far larger than the 2 g/day proposed here [56].

The clinical meaning of these findings remains contested. The fasting association cannot distinguish dietary exposure from endogenous xylitol production or fully exclude residual metabolic confounding, and the human challenge had no matched control and used 30 g at one time, fifteen times the 2 g daily amount proposed in this formulation. Published critiques have therefore questioned whether the results can be extrapolated to low-dose, divided oral-health use [57,58]. Witkowski and Hazen responded that they observed platelet effects at lower experimental concentrations and emphasized that long-term cardiovascular-outcome studies of dietary xylitol have not been performed [59]. Accordingly, 2 g/day should not be described as cardioprotective or presumed to be free of cardiovascular risk. Its chronic effects on platelet function and clinical cardiovascular outcomes are unknown, and dose-specific prospective studies are needed.

The bidirectional association between diabetes and periodontitis further supports monitoring host metabolic status when studying oral inflammation [60]. Glycemic efficacy is outside the primary scope of this review.

### 9.3. The Oral–Gut Axis, Barrier Function, and Intestinal Inflammation

Most swallowed oral bacteria do not establish permanent residence in a healthy colon, but niche separation is not absolute. Gastric acid suppression, antibiotics, periodontal disease, intestinal inflammation, impaired colonization resistance, and mucosal injury can increase oral-to-gut transmission. Experimental work has shown that oral organisms capable of ectopic intestinal colonization can drive T helper type 1 (T_H_1) responses and intestinal inflammation in susceptible hosts [61]. This does not mean that every oral organism swallowed each day becomes a gut pathogen. It does mean that reducing the upstream oral burden and maintaining a resistant intestinal community are complementary preventive strategies.

Large-scale, strain-level metagenomic surveys have since extended this evidence base, showing that oral-to-gut microbial transmission occurs to some degree in everyone but is markedly amplified in inflammatory bowel disease (IBD) and cirrhosis, where it contributes disproportionately to gut dysbiosis [62]. Direct experimental confirmation comes from murine work showing that oral infection with a periodontal pathogen measurably alters the composition of the distal gut microbiome, not only the oral community, indicating a compositional link rather than a simple aspiration artifact [63]. Reviews of this oral–gut axis converge on a similar mechanism: loss of gut colonization resistance, whether from antibiotics, acid suppression, or inflammation, allows swallowed oral organisms such as *P. gingivalis*, *F. nucleatum*, and oral Enterobacteriaceae to establish ectopically and to sustain gut inflammation [64,65]. In inflammatory bowel disease specifically, expansion of oral-origin pathobionts in the gut has been proposed as both a marker and a driver of disease activity [66]. These findings strengthen, rather than replace, the original rationale: reducing the oral reservoir and supporting intestinal colonization resistance are complementary strategies against the same translocation route.

The four-strain formulation addresses both sides of this route. M18 and xylitol act at the oral source; HEAL9 and 8700:2 provide gut-level immune signaling; and *B. infantis*, when paired with an appropriate substrate and host context, supports bifidobacterial metabolism, lower luminal pH, acetate and lactate production, epithelial barrier function, and competitive exclusion [6,7,32], consistent with the symptom and cytokine-profile benefits reported for related lactobacilli and bifidobacteria in other gut disorders such as irritable bowel syndrome [67]. The formulation may therefore reduce the number and inflammatory activity of pathobionts entering the gut while strengthening resistance at the site of entry. This dual-site model is more defensible than claiming that an oral probiotic permanently colonizes every compartment.

### 9.4. The Oral–Nasal–Pulmonary Continuum and the Gut–Lung Axis

The lower airway was long assumed to be sterile in health, but culture-independent sequencing has shown that the healthy lung harbors a low-biomass community that is not self-sustaining; rather, it is continuously seeded by subclinical microaspiration of oral secretions and cleared by mucociliary and immune activity, a relationship formalized as the ‘adapted island’ model of the lung microbiome [68]. Direct topographic sampling supports this model: bacterial community composition changes gradually and continuously from the mouth to the lower airway in healthy adults, with lower-airway communities resembling a diluted, selected subset of the oral community rather than a distinct one [69]. Because microaspiration carries material from the oral and oropharyngeal reservoir, an intervention that reduces oral pathobiont burden has a plausible, if indirect, route to influencing the composition of what is seeded into the lower airway even in the absence of overt aspiration disease.

The mouth and nose also serve as reservoirs for organisms that reach the lower airway through aspiration. This route is especially relevant in older adults, hospitalized patients, individuals with dysphagia, patients receiving enteral feeding, and those with poor oral hygiene or heavy dental plaque. Lowering the oral and nasopharyngeal pathogen burden is therefore potentially relevant to respiratory health even when the probiotic does not directly colonize the lung. The HEAL9/8700:2 trials and the broader Cochrane analysis support a possible reduction in upper respiratory infection burden with sustained probiotic use, while the xylitol literature supplies a separate airway-surface and anti-adhesion mechanism [4,5,9,30,42,43,44,45].

A separate but convergent pathway is the gut–lung axis. Gut microbial metabolites and gut-trained immune cells influence pulmonary immune tone at a distance, such that gut dysbiosis is associated with increased susceptibility to respiratory infection, asthma, and COPD exacerbation [70]. Because *B. infantis*, HEAL9, and 8700:2 act substantially at the gut level, their contribution to respiratory outcomes plausibly runs through this axis as well as through the oropharyngeal route described above. Upper-airway ecology also predicts downstream respiratory risk beyond the infancy window already discussed in Section 3: nasopharyngeal microbiota maturation trajectories in the first months of life predict subsequent respiratory tract infection risk [71], and a critical developmental window has been identified in which the interplay between nasal and bronchial microbiota and specific pathogenic bacteria precedes childhood asthma-related respiratory disease [72]. Table 3 summarizes this expanded evidence base linking the oral and nasal microbiome to gut and pulmonary outcomes.

### 9.5. Maternal–Child Transmission and Early-Life Ecology

Maternal xylitol trials show reduced or delayed transmission of mutans streptococci to children [37,38]. This finding supports study of the maternal oral reservoir and infant colonization within the gateway model; it does not establish obstetric or developmental benefits for the proposed formulation.

Cannon and Peldyak described pediatric oral–systemic health as a continuum beginning with the maternal microbiome and extending through infancy, childhood, and adolescence [73]. An oral–nasal synbiotic is therefore best studied longitudinally, with age-appropriate endpoints rather than as an isolated adult supplement.

The early-life role of *B. infantis* is also inseparable from human milk oligosaccharides [6,7,32]. A strain may be biologically capable of HMO utilization, but the expected ecological effect depends on breastfeeding, HMO availability, dose, viability, antibiotic exposure, and the resident microbiome. In premature or critically ill infants, use requires direct clinical oversight because probiotic organisms can rarely cause invasive infection in highly vulnerable hosts [74].

## 10. Safety, Dosing, and Populations

Safety findings from component trials cannot establish the safety of the complete formulation. Pregnancy, infancy, immunocompromise, and critical illness require population-specific assessment and clinical oversight [74].

Xylitol is generally well tolerated at dental doses. High intake can cause transient gastrointestinal symptoms such as bloating or osmotic diarrhea, which are dose-dependent and may diminish with adaptation [75,76]. At the proposed 2 g/day, the formulation sits far below the intake associated with significant gastrointestinal effects in adults.

Vitamin C and vitamin D3 amounts should be specified before clinical testing and assessed against age-appropriate intake limits. Dose, administration frequency, delivery vehicle, and duration should be prespecified; component trials do not establish an effective regimen for the combined formulation.

## 11. Level of Evidence for the Proposed Benefits

**Figure 6:**
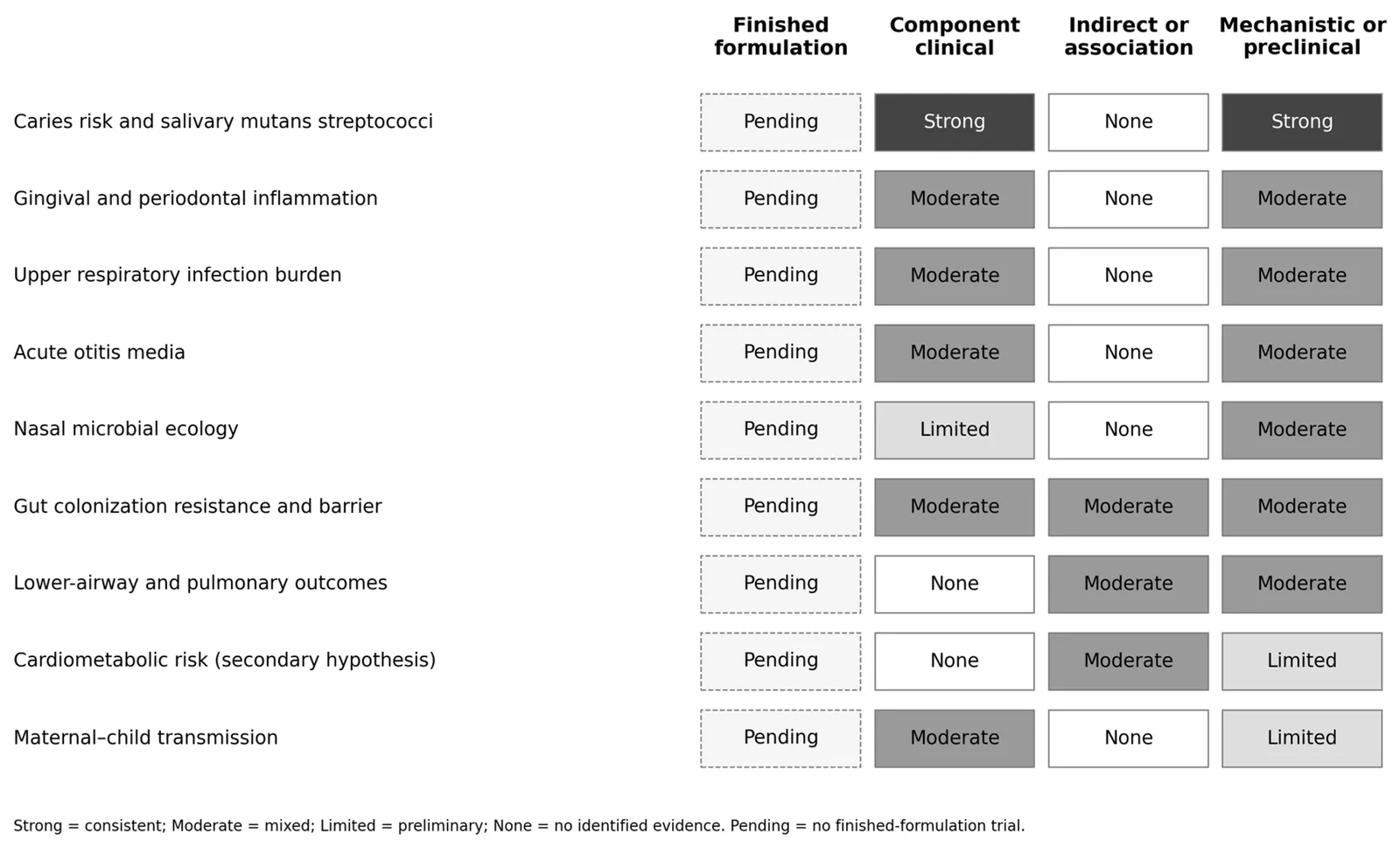
Graphical summary of evidence strength for each proposed benefit. Columns separate finished-synbiotic evidence, component-level clinical evidence for the outcome itself, indirect clinical or associational evidence, and mechanistic or preclinical evidence. Shading indicates strong and consistent, moderate or mixed, limited or preliminary, or no identified evidence. This qualitative assessment summarizes Table 5. “Pending” marks outcomes awaiting a trial of a synbiotic formulation.

Table 4 defines the evidence tiers used in this review, Table 5 applies them to each proposed benefit, and Figure 6 presents the same assessment graphically. No outcome is supported by Tier 1 evidence because the complete formulation has not been tested. The strongest component-level support is for caries-related outcomes (M18 and xylitol) and for upper respiratory infection burden (HEAL9/8700:2 and vitamin D). Clinical evidence for gut benefits is mainly from breastfed infants. Pulmonary, cardiometabolic, and pregnancy-related benefits rest on indirect or mechanistic evidence, apart from the effect of maternal xylitol on transmission of mutans streptococci.

## 12. Discussion

Three main topics underpin this review. First, the oral and nasal compartments form a connected ecosystem with distinct local niches, renewed by swallowing and mucociliary clearance on a time scale of minutes (Section 3.1); a formulation intended for this gateway should therefore be judged by its effect on the connected system, not on any one site in isolation. Second, the components of the described synbiotic are complementary rather than redundant: direct bacteriocin-mediated antagonism from *S. salivarius* M18 and metabolic interference from xylitol act on a largely non-overlapping set of organisms compared with the host-mediated, ecological contributions of the lactobacilli, *B. infantis*, and the two vitamins (Section 6), which is the mechanistic basis for combining them rather than selecting a single ingredient. Third, the downstream consequences of gateway ecology extend further than the mouth and nose: swallowed oral organisms can seed the gut through loss of colonization resistance (Section 9.3), and the lower airway is continuously seeded by subclinical microaspiration of oral secretions while gut-derived signals train pulmonary immunity through the gut–lung axis (Section 9.4).

These mechanisms support further research rather than expanded clinical claims. Nearly every individual mechanism reviewed here, bacteriocin production, xylitol’s futile metabolic cycle, vitamin D-induced cathelicidin, oral-to-gut translocation, and lung seeding by microaspiration, is well supported in isolation. What is not yet established is that the specific four-strain, xylitol, and vitamin combination described here reproduces these effects as a finished formulation, at the stated doses, over a clinically meaningful period. This review deliberately maintains the distinction between component-level plausibility and product-level proof, considering the formulation’s oral–systemic, gut, or pulmonary potential as hypothesized.

### 12.1. Areas of Consensus and Controversy

Some conclusions in this review rest on broad agreement. Strain M18 reduces plaque indices and salivary mutans streptococci in children [2,27,28], xylitol lowers salivary mutans streptococci in a dose-dependent manner [36], and the HEAL9/8700:2 pair reduced common cold burden in two controlled trials [4,5]. The physiological basis of oral–nasal exchange, including swallowing frequency and mucociliary transit, is also well established [23,24]. Other points remain contested. Xylitol for acute otitis media illustrates the problem: an early randomized trial reported fewer episodes with xylitol gum [42], a Cochrane review judged the overall evidence to be of limited certainty [77], and a 2024 trial in children aged 1 to 5 years found no benefit [43]. The cardiovascular signal attached to circulating xylitol is a second area of active disagreement (Section 9.2) [56,57,58,59]. The periodontal probiotic literature shows the same pattern in another form, with favorable pooled estimates but substantial heterogeneity by strain, dose, and population [52,53,54].

### 12.2. From Single Strains to a Multi-Strain Formulation

The most important interpretive limit is the step from single-strain evidence to a multi-strain product. A systematic review of probiotic trials found that efficacy was both strain-specific and disease-specific, and that pooling different strains obscured real differences [78]. Reviews comparing mixtures with single strains report that mixtures sometimes perform better, but direct comparisons are uncommon, and the reasons for any advantage are seldom identified [79,80]. Strains in one product can compete for adhesion sites or nutrients, and their doses, viability, and release may differ from those used in the original trials. Host factors add further variation: mucosal colonization by ingested probiotics differs markedly between individuals and depends on the resident microbiome [81], and probiotic use after antibiotics can delay recovery of the native gut community [82]. For the present formulation, M18 results from oral lozenge or tablet preparations, HEAL9/8700:2 results from a two-strain preparation, and B. infantis results from breastfed infants cannot be assumed to hold when the strains are combined with each other, with xylitol, and with vitamins in one product. Each of these assumptions is testable, and a factorial or component-dismantling design would show whether the combination adds benefit beyond its most active ingredients.

### 12.3. Comparison with Existing Reviews and Competing Strategies

Published reviews have generally addressed one compartment at a time: oral probiotics as adjuncts in caries and periodontal care [49,53], the upper and lower respiratory tracts as a microbial ecosystem [15,26], and the oral–gut axis as a route of translocation in intestinal disease [64,65]. The present review differs in two respects. It treats the nasal, oral, and gut compartments as one exchange system linked by swallowing and mucociliary clearance, and it evaluates a specific combination against that model rather than surveying probiotics in general. Competing strategies exist for each compartment. For the oropharynx, *S. salivarius* K12 has been studied to prevent recurrent streptococcal and viral pharyngotonsillitis in children [83]. For the nose, xylitol can be delivered directly by irrigation or spray, and a pilot study in chronic rhinosinusitis reported greater symptom improvement with xylitol irrigation than with saline [84]. For the gut, single-strain and consortium products have stronger indication-specific evidence than any multi-compartment product. An oral synbiotic is not a substitute for these approaches. Its possible advantage is simultaneous action across compartments, and whether that advantage exists in practice has not been shown.

### 12.4. Translational Challenges

Several practical issues stand between the mechanistic model and clinical use. Oral probiotics must survive manufacture, storage, and passage through the mouth in sufficient numbers, and commercial products do not always contain the organisms or counts stated on the label [85]. Exposure differs by compartment: an oral product contacts the oropharynx directly but reaches the nasal mucosa only indirectly, which is why Section 7 discusses direct nasal delivery of xylitol separately. Outcome measurement is also difficult. Microbial communities vary within the same person over time, and a change in relative abundance does not by itself show a change in disease risk [86]. Finally, trials of microbiota-modifying interventions often incompletely report harms [87], and the safety of a multi-ingredient product in pregnancy, infancy, and immunocompromised patients requires specific evaluation rather than extrapolation from its components [74].

## 13. Limitations and Future Directions

### 13.1. Limitations of the Current Evidence

The main limitation is that no randomized clinical trial has tested the complete formulation, four strains with 2 g of xylitol and vitamins C and D3, as a finished product. All support for the combination is therefore indirect, drawn from single-agent or two-strain trials, class-level meta-analyses, and mechanistic studies (Table 5).

Second, probiotic effects vary by strain, dose, delivery vehicle, duration, baseline disease, diet, and adherence, and published trials differ in population, comparator, and endpoint. This heterogeneity limits pooling and makes product-specific validation preferable to category-level reasoning [53,54,78].

Third, causality is difficult to establish across microbiome axes. Associations between oral taxa and gut, lung, or cardiovascular outcomes can reflect shared risk factors, reverse causation, or disease-related changes in sampling, and animal models do not always predict human effects [88]. The temporal fluid-dynamic model in Section 3.1 and the oral–gut and oral–nasal–pulmonary pathways in Section 9.3 and Section 9.4 are consistent with general physiology [68,69,70,89] but have not been tested with this proposed synbiotic formulation.

Fourth, nasal applications of probiotics and xylitol are supported by mechanistic studies and early clinical data but lack the large, standardized trials available for oral and gut outcomes [9,29,84].

Fifth, regulatory status limits what can be claimed. In the United States, a product of this type would generally be marketed as a dietary supplement, which permits structure and function statements but not claims to diagnose, treat, cure, or prevent disease. The statements in this review about respiratory, gut, pulmonary, and systemic outcomes are research hypotheses and should not be read as product claims. Verified strain identity, viable counts, and stability over shelf life are further requirements [85].

Finally, this is a structured narrative review rather than a systematic review. Study selection and evidence grading involved author judgment, and relevant studies may have been missed.

### 13.2. Future Directions

Four priorities follow. First, a randomized, placebo-controlled trial of the finished product should use caries-risk and gingival indices and upper respiratory infection burden as primary endpoints, because these outcomes have the strongest component-level support. A factorial design, or at minimum an arm containing M18 and xylitol alone, would show whether the additional strains and vitamins add benefit. Second, longitudinal sampling of saliva, the anterior nares or nasopharynx, and stool, with lower-airway sampling where feasible, would test whether the product changes microbial exchange between compartments rather than at a single site. Third, multi-omics methods, including strain-resolved metagenomics, metatranscriptomics, and metabolomics, can link changes in community structure to function and to host inflammatory markers [86]. Fourth, trials should report harms systematically [87] and include prespecified subgroups such as pregnant women, children, and older adults at risk of aspiration. The initial research program should remain focused on oral, nasal, intestinal, and respiratory endpoints.

## 14. Conclusions

The oral–nasal gateway is a connected system, renewed on a time scale of minutes by swallowing and mucociliary clearance (Section 3.1), and a formulation designed for it should be evaluated across compartments. *S. salivarius* M18 supplies direct antagonism in the mouth and oropharynx; *L. plantarum* HEAL9 and *L. paracasei* 8700:2 supply immune-mediated respiratory support; *B. infantis* anchors gut colonization resistance and early immune imprinting; xylitol adds anticariogenic and airway-active mechanisms; and vitamins C and D3 reinforce host defense. Oral-probiotic studies support sustained, monitored ecological therapy [33,34,35], while a published life-course model places intervention from pregnancy through adolescence [73]. The oral–gut and gut–lung axis literature extends this logic downstream, linking gateway ecology to intestinal and pulmonary outcomes through translocation and immune-training routes that are well documented in general microbiome science but not yet tested for this specific formulation [62,63,64,65,66,68,69,70]. At present, the strongest support is for caries-related and upper respiratory outcomes at the component level; gut, pulmonary, and systemic benefits remain hypotheses. A randomized trial of a finished formulation with multi-compartment sampling is the necessary next step.

## Figures and Tables

**Table 1:** Formulation overview. CFU, colony-forming units; BLIS, bacteriocin-like inhibitory substances; RDA, recommended dietary allowance. Amounts are representative; verify against the proposed synbiotic specification.

Component	Amount	Primary Compartment	Principal Documented Role
*S. salivarius* M18	≥1 × 10^9^ CFU	Oral/oropharynx	BLIS-mediated antagonism of cariogenic, periodontal, and pyogenic streptococci
*L. plantarum* HEAL9	≥1 × 10^9^ CFU	Gut/systemic immune	Immune modulation; reduced common cold burden (with 8700:2)
*L. paracasei* 8700:2	≥1 × 10^9^ CFU	Gut/systemic immune	Immune modulation; upper respiratory support (with HEAL9)
*B. infantis*	≥1 × 10^9^ CFU	Gut	Colonization resistance; early-life immune imprinting; barrier support
Xylitol	2 g	Oral + nasal	Anticariogenic; reduces nasal bacterial adhesion (airway osmolyte)
Vitamin C	typical RDA–upper	Systemic/gingival	Innate and epithelial immune support; collagen and gingival integrity
Vitamin D3	typical RDA–upper	Systemic/mucosal	Immune modulation; cathelicidin induction; periodontal and respiratory support

**Table 2:** Pathogen-by-pathogen summary of active components and documented mechanisms. See Figure 2 for the full matrix.

Pathogen	Active Component(s)	Mechanism	Clinical Relevance
*S. mutans*	M18; xylitol	BLIS; non-fermentable futile cycle; reduced adhesion	Dental caries
*S. sobrinus*	M18; xylitol	BLIS; acid-suppression	Dental caries
*S. pyogenes*	M18	Salivaricin bacteriocins	Pharyngitis/tonsillitis
*P. gingivalis*	M18; xylitol; vitamin D3	Antagonism; reduced immune activation; host defense	Periodontitis; oral–systemic
*A. actinomycetemcomitans*	M18; vitamin D3	Antagonism; host defense	Aggressive periodontitis
*C. albicans*	M18; xylitol; lactobacilli	Competition; reduced adhesion; ecology	Oral candidiasis
*S. pneumoniae*	Xylitol; lactobacilli (indirect)	Reduced adhesion/growth; immune modulation	Otitis media; respiratory infection
*H. influenzae*	Xylitol	Reduced nasopharyngeal growth/adhesion	Otitis media; sinusitis
*M. catarrhalis*	Xylitol	Reduced adhesion	Otitis media; sinusitis
*S. aureus*	Xylitol; vitamin D3	Airway-surface killing; cathelicidin	Nasal carriage; respiratory disease
Enteric pathobionts	*B. infantis*; lactobacilli	Colonization resistance; barrier support	Gut dysbiosis; systemic inflammation

**Table 3:** Selected evidence linking the oral and nasal microbiome to gut and pulmonary outcomes.

Axis	Source	Design/Model	Key Finding
Oral→gut translocation	Atarashi et al. [61]	Mouse model; oral pathobiont gavage	Oral bacteria ectopically colonize the inflamed gut and drive T_H_1 induction and colitis
Strain-level transmission	Schmidt et al. [62]	Human metagenomic cohort	Oral-to-gut microbial transmission occurs universally and is amplified in IBD and cirrhosis
Oral pathogen–gut link	Simas et al. [63]	Mouse model; *P. gingivalis* oral infection	Oral infection alters both the oral and the distal gut microbiome
Oral–gut axis synthesis	Read et al. [64]; Kunath et al. [65]; Wang et al. [66]	Narrative/systematic reviews	Synthesize translocation routes and IBD associations of oral-origin pathobionts
Lung seeding model	Dickson et al. [68]	Conceptual model + cohort review	Healthy lung microbiome is continuously seeded by subclinical microaspiration (‘adapted island’ model)
Topographic continuity	Dickson et al. [69]	Human bronchoscopy cohort	Bacterial composition changes continuously from mouth to lower airway in health
Gut–lung axis	Budden et al. [70]	Review	Gut microbial metabolites and immune training influence asthma/COPD susceptibility
Infant nasopharyngeal maturation	Teo et al. [22]; Bosch et al. [71]	Infant cohort studies	Nasopharyngeal microbiota trajectories predict respiratory infection and asthma risk
Airway microbiota window	Teo et al. [72]	Infant cohort	A critical developmental window links nasal/bronchial microbiota interplay to childhood respiratory disease
Upper-airway ecosystem model	Man et al. [26]	Review	Frames the nasal, oral, and lower airway as one continuous ecosystem

**Table 4:** Evidence hierarchy for the proposed oral–nasal synbiotic, including explicit evidence tiers.

Tier	Evidence Category	Examples in This Review	Permitted Interpretation
1	Direct finished-synbiotic evidence	None published for the complete four-strain formulation with 2 g of xylitol and vitamins C and D3	Efficacy claim for the finished synbiotic
2	Component- or strain-specific clinical evidence	M18 oral outcomes; HEAL9/8700:2 respiratory outcomes; xylitol dental and otitis outcomes; B. infantis infant-gut studies	Supports component selection; does not establish the effect of the combination
3	Mechanistic and translational evidence	Bacteriocins, xylitol antimicrobial and airway effects, oral-to-gut translocation, microaspiration, and possible oral–systemic benefit	Defines hypotheses and endpoints; does not establish clinical benefit or harm with direct evidence

**Table 5:** Level of evidence for each proposed benefit of the oral–nasal synbiotic. Tiers follow Table 4. Tier 1 (finished-synbiotic) trials are not yet available for any outcome, so that tier is not repeated in the table.

Proposed Benefit	Best Available Evidence	Key Sources	Principal Limitation
Lower caries risk and salivary mutans streptococci	Tier 2: randomized trials of M18 in children; xylitol dose–response and maternal-transmission trials and meta-analysis	[2,27,28,36,37,38]	Component trials only; mostly surrogate endpoints (plaque, salivary counts, Cariogram)
Less gingival and periodontal inflammation	Tier 2 at class level: adjunctive probiotic meta-analyses; Tier 3: M18 cytokine suppression in fibroblasts; xylitol activity against *P. gingivalis*	[40,41,51,52,53,54]	High heterogeneity; M18 not tested in periodontitis trials
Fewer or shorter upper respiratory infections	Tier 2: HEAL9/8700:2 trials; vitamin D and vitamin C meta-analyses; class-level Cochrane review	[4,5,10,30,47]	Strain pair tested without M18, xylitol, or *B. infantis*; certainty low to moderate
Prevention of acute otitis media	Tier 2, mixed: positive xylitol gum trial; null 2024 trial	[42,43]	Doses and regimens differ from 2 g/day; inconsistent results
Healthier nasal microbial ecology	Tier 3 with early clinical data: airway surface liquid and in vitro xylitol studies; nasal lactobacilli	[9,29,44,45]	Awaits a trial of the oral synbiotic measuring nasal community change
Gut colonization resistance and barrier support	Tier 2 in breastfed infants (*B. infantis* EVC001); Tier 3 for oral-to-gut translocation	[6,7,32,61,62,63,64,65,66]	Strain-, age-, and substrate-specific; adult relevance unknown
Lower-airway and pulmonary benefit	Tier 3: lung seeding by microaspiration; gut–lung axis	[68,69,70]	No clinical data for any component on lower-airway outcomes
Cardiometabolic risk	Tier 3 (association): periodontitis–cardiovascular and diabetes–periodontitis consensus reports	[55,60]	Periodontal treatment not shown to prevent events; xylitol platelet signal unresolved [56,57,58,59]
Maternal–child transmission and early life	Tier 2 for transmission of mutans streptococci (maternal xylitol); other outcomes untested	[37,38,73]	Obstetric, allergic, and developmental outcomes not studied

## Data Availability

No new data were created or analyzed in this review. Data sharing is not applicable to this article.

## Abbreviations

The following abbreviations are used in this manuscript:

Abbreviation	Definition
BLIS	Bacteriocin-like inhibitory substance(s)
CFU	Colony-forming unit(s)
COPD	Chronic obstructive pulmonary disease
CRT	Caries Risk Test
HMO	Human milk oligosaccharide(s)
IBD	Inflammatory bowel disease
IL	Interleukin
RDA	Recommended dietary allowance
T_H_1	T helper type 1 (lymphocyte subset)
